# Prevalence of tobacco product use and associated factors among healthcare workers in Trabzon, Türkiye: A cross-sectional study

**DOI:** 10.18332/tid/224356

**Published:** 2026-08-08

**Authors:** Muhammed İ. Özel, Serdar Karakullukçu

**Affiliations:** 1 Department of Public Health, Faculty of Medicine, Karadeniz Technical University, Trabzon, Türkiye

**Keywords:** healthcare workers, tobacco use, nicotine dependence, novel nicotine and tobacco products

## Abstract

**INTRODUCTION:**

Tobacco use among healthcare workers is important not only in terms of individual health risks but also regarding the effectiveness of tobacco control services. The aim of this study was to determine the prevalence of tobacco use among healthcare personnel working in public primary, secondary, and tertiary care facilities in Trabzon Province and to assess factors associated with tobacco use.

**METHODS:**

This cross-sectional study was conducted between July 2025 and February 2026 among healthcare workers aged ≥18 years who were actively employed in public healthcare institutions in Trabzon Province and agreed to participate. Data were collected from 1381 participants through face-to-face interviews. Use of any tobacco product within the past 30 days was considered the primary dependent variable. Nicotine dependence was assessed using the Fagerström test for nicotine dependence (FTND). Logistic regression analysis was performed to identify factors associated with tobacco use and nicotine dependence.

**RESULTS:**

The prevalence of current tobacco product use was 25.2% (95% CI: 23.0–27.4). Specifically, 20.9% used only traditional tobacco, 1.5% used only novel nicotine and tobacco products, and 2.8% were dual users. Approximately half of the users exhibited moderate-to-high nicotine dependence. Multivariable analysis showed current use was strongly associated with having a tobacco user in the close environment (AOR=10.40; 95% CI: 5.20–20.81). Male sex (AOR=1.84; 95% CI: 1.33–2.56), shift/on-call work (AOR=1.51; 95% CI: 1.12–2.04), exposure to pro-tobacco social media content (AOR=1.73; 95% CI: 1.31–2.27), and higher stress scores (AOR=1.31; 95% CI: 1.22–1.42) were also associated with current use. Among users, male sex increased the odds of moderate-to-high nicotine dependence (AOR=2.04; 95% CI: 1.23–3.38), whereas living with family was associated with lower odds (AOR=0.47; 95% CI: 0.24–0.92).

**CONCLUSIONS:**

Tobacco use among healthcare workers remains a significant public health issue. These findings reveal associations between tobacco use and individual, occupational, and psychosocial factors, highlighting the need for longitudinal studies and tailored tobacco control strategies in this population.

## INTRODUCTION

Tobacco use is a major cause of preventable morbidity and mortality, resulting in more than 7 million deaths worldwide each year^1^. Tobacco use exerts a considerable impact on both individual and public health, given its association with various health complications, including cardiovascular diseases, chronic respiratory diseases, malignancies, and numerous other chronic conditions^2^. Therefore, tobacco control is regarded as a fundamental public health priority that should not be limited to policies targeting the general public but should also encompass healthcare workers within the healthcare system^3^.

As with the general population, tobacco use among healthcare workers remains a significant public health problem. International data indicate that the prevalence of tobacco use among healthcare professionals is still at considerable levels^4^. Despite high levels of knowledge and awareness regarding the harmful effects of tobacco on health, factors such as heavy workload, shift work, on-call systems, and high occupational stress have been reported to contribute to the initiation and continuation of tobacco use among healthcare workers^4,5^.

The use of tobacco among healthcare professionals constitutes not only an individual health risk, but also an important impediment to the success of tobacco control policies in the broader societal context^6,7^. Healthcare professionals serve a dual role in public health, functioning as both a reliable source of information and a model for healthy lifestyle behaviors. It has been documented that healthcare professionals who utilize tobacco products themselves may exhibit a reduced propensity to offer smoking cessation counseling to their patients and may harbor more unfavorable attitudes toward the issue^6,7^. Consequently, the reduction of tobacco use among healthcare professionals represents a pivotal strategy in addressing the broader tobacco epidemic.

In recent years, the emergence of novel nicotine and tobacco products, including electronic cigarettes (e-cigarettes) and heated tobacco products (HTPs), has profoundly impacted the landscape of global tobacco epidemiology, alongside the ongoing prevalence of traditional cigarettes^8^. Despite the tobacco industry’s frequent marketing of these products as ‘less harmful’ alternatives, they pose significant risks to the cardiovascular and respiratory systems due to the toxic substances and nicotine they contain^9,10^. These products, which garner attention due to their innovative designs and assertive marketing strategies on social media, are also being used with increasing frequency among healthcare professionals. In addition, the ‘dual-use’ model, in which both traditional and novel nicotine and tobacco products are used simultaneously, poses a modern and complex threat to tobacco control by increasing exposure to toxins^10^.

Despite the implementation of MPOWER strategies in tobacco control at the highest level, Türkiye still exhibits a high prevalence of tobacco use among those aged ≥15 years (28.3%)^11,12^. A review of the national literature reveals that extant research on tobacco use among healthcare professionals is generally single-centered, limited to specific hospitals or a single professional group (only physicians or only nurses)^13-16^. Additional comprehensive data may help to better characterize tobacco product use across various professional groups (e.g. physicians, nurses/midwives, and other healthcare personnel) working in primary, secondary, and tertiary healthcare institutions. Such data may also help clarify the current prevalence of traditional and novel nicotine and tobacco products, including dual use, alongside emerging exposure-related factors such as social media exposure. This study contributes to the national literature by evaluating traditional and novel nicotine and tobacco product use, dual use, nicotine dependence, cessation-related characteristics, and social media exposure together among healthcare workers across primary, secondary, and tertiary public healthcare institutions at the province level. In this context, the objective of this study is to ascertain the prevalence of tobacco product use among healthcare personnel working in public primary, secondary, and tertiary healthcare institutions in Trabzon province and to evaluate the sociodemographic, work-related, and environmental factors associated with this behavior.

## METHODS

### Design of the study

This cross-sectional study was conducted between July 2025 and February 2026 in public health institutions located in the province of Trabzon, northeastern Türkiye. The study population was defined to include primary, secondary, and tertiary care facilities in order to comprehensively reflect the structure of the health system across the province.

### Study population and sample

The target population of the study consists of a total of 9520 healthcare workers, including physicians, nurses/midwives, and other healthcare personnel, who were employed in public primary, secondary, and tertiary healthcare institutions in Trabzon province during the study period from July 2025 to February 2026. The OpenEpi program^17^ was used to determine the sample size. Based on the results of a 2019 systematic review and meta-analysis investigating tobacco use prevalence among healthcare workers^4^, the expected prevalence of tobacco product use was calculated as 1255 individuals, with a 95% confidence interval and precision value of 2.1% (one-tenth of the expected prevalence). Taking into account potential data losses and non-response, a 10% margin of error was added to the sample size, and the final sample was determined to be 1381 individuals. The selection of the sample was executed through the implementation of stratified sampling. The target population was stratified according to the level of the healthcare facility (primary, secondary, and tertiary care) and profession (physicians, nurses/midwives, and other healthcare personnel). In order to enhance the representativeness of the sample with respect to the population, the number of individuals to be selected from each stratum was weighted proportionally to their distribution in the population.

### Inclusion and exclusion criteria

Healthcare workers aged ≥18 years who were actively employed in public primary, secondary, or tertiary healthcare institutions in Trabzon province during the study period and who agreed to participate, were included in the study. Physicians, nurses/midwives, and other healthcare personnel were eligible for inclusion. Healthcare workers who were on long-term leave during data collection, could not be reached after repeated contact attempts, declined to participate, or provided incomplete questionnaire data for the primary outcome variable, were excluded from the study.

### Ethics

Approval for this study was obtained from the Scientific Research Ethics Committee of the Faculty of Medicine at Karadeniz Technical University (Decision No.: 2025/180; Date: 2 July 2025). The necessary institutional permissions for conducting the study were obtained from the Trabzon Provincial Health Directorate and relevant healthcare institutions. The research was conducted in accordance with the principles of the Declaration of Helsinki. Prior to data collection, the purpose of the study was explained to the participants, assurance was provided that the collected data would remain anonymous, and verbal consent was obtained from those who agreed to participate in the study.

### Data collection tools

Data were collected through face-to-face interviews using a 63-item structured questionnaire developed by the researchers based on a literature review (The questionnaire is provided in the Supplementary file). The questionnaire assessed participants’ sociodemographic characteristics (age, gender, marital status, education level, income, living arrangements), health status (chronic diseases, psychiatric disorders), work-related characteristics (profession, workplace, job level, duration of employment, shift work status), tobacco product usage habits (usage status, type, age of initiation, duration, quantity, and cessation experiences), social media exposure (daily usage time, frequency of encountering promotional content, and its impact), the presence of tobacco users in their close environment, and stress levels.

*Dependent variables*

The primary dependent variable of the study is the use of any tobacco product (traditional cigarettes, hand-rolled cigarettes, hookah, cigars, pipes, heated tobacco products, e-cigarettes) within the past 30 days. Users were classified into three groups: those who use only traditional tobacco products, those who use only novel nicotine and tobacco products, and those who use both (dual users).

The secondary dependent variable is the level of nicotine dependence, measured using the Fagerström test for nicotine dependence (FTND) exclusively among smokers. The FTND consists of six questions and is scored on a scale of 0–10; scores of 0–3 are classified as low, 4–6 as moderate, and 7–10 as high dependence^18^. The reliability and factor structure of the Turkish version of the FTND were evaluated by Uysal et al.^19^. In this study, the dependence level was categorized as low (0–3) and moderate/high (4–10) for binary logistic regression.

To measure participants’ perceived stress levels over the past month, the general stress level (0–10 visual analog scale) was used. The general stress level is an assessment based on self-report, in which high scores indicate high stress.

### Statistical analysis

Statistical analyses were performed using the SPSS 26.0 (SPSS, IBM Corp, Armonk, New York, NY, USA)^20^ software package. Descriptive statistics are presented as frequencies and percentages, means ± standard deviations, or medians (minimum–maximum). In univariate analyses, the chi-squared test, Student’s t-test, and Mann-Whitney U test were used depending on the nature and distribution characteristics of the variables. Logistic regression analysis was applied to evaluate factors independently influencing the use of any tobacco product. Additionally, logistic regression analysis was conducted to assess the factors influencing the level of nicotine dependence among participants who use tobacco products. Unadjusted associations were presented as odds ratios (ORs) with 95% confidence intervals in the Supplementary file tables, whereas multivariable-adjusted associations were presented as adjusted odds ratios (AORs) with 95% confidence intervals in the main tables and text. Variables with a p<0.25 in univariate analyses were considered candidate variables for inclusion in the multivariable logistic regression models. Multicollinearity among candidate variables was assessed before model construction; when multicollinearity was detected between variables, only one of the correlated variables was retained in the model. For the multivariable logistic regression model assessing factors associated with current tobacco product use, the dependent variable was current use of any tobacco product within the past 30 days. The covariates included in this model were age, gender, marital status, have children, education level, income status, living arrangement, profession, level of care, work schedule, presence of at least one tobacco user in the close environment, psychiatric disease, exposure to pro-tobacco content on social media, and self-reported stress score. For the multivariable logistic regression model assessing factors associated with moderate-to-high nicotine dependence among current tobacco users, the dependent variable was moderate-to-high nicotine dependence, defined as an FTND score of 4–10. The covariates included in this model were age, gender, income status, living arrangement, psychiatric disease, age at first tobacco product use, and self-reported stress score. The forest plot of the logistic regression model used to evaluate the risk factors for nicotine dependence was visualized using the Python version 3.12 (Python Software Foundation, Wilmington, DE, USA)^21^. A p<0.05 was considered statistically significant.

## RESULTS

A total of 1381 healthcare workers were included in the study. The mean age of the participants was 38.8 ± 9.6 years, and the mean total years of work experience was 15.3 ± 9.8. Most participants were female (70.8%), married (72.2%), and had children (65.2%). More than half had a college degree (58.3%), and nearly half reported that their income was equal to their expenses (49.5%). Nurses/midwives constituted the largest professional group (38.9%), followed by other healthcare personnel (37.8%) and physicians (23.3%). Nearly half of the participants worked in tertiary care institutions (49.6%), and 44.0% were engaged in shift/on-call work. Chronic disease and psychiatric disease were reported by 27.4% and 9.1% of the participants, respectively (Table 1). More than half of the participants (51.6%) reported exposure to pro-tobacco content on social media, 88.3% had at least one tobacco user in their close environment, and the mean self-reported stress score was 5.8 ± 1.8.

**Table 1:** Baseline characteristics of healthcare workers in a cross-sectional study conducted in public primary, secondary, and tertiary care facilities in Trabzon, Türkiye, July 2025–February 2026 (N=1381)

*Characteristics*	*Categories*	*n*	*%*
**Age** (years), mean ± SD		38.8 ± 9.6	
**Work experience** (years), mean ± SD		15.3 ± 9.8	
**Gender**	Female	978	70.8
	Male	403	29.2
**Marital Status**	Married	997	72.2
	Single	384	27.8
**Have children**	Yes	901	65.2
	No	480	34.8
**Education level**	High school /Associate’s degree	190	13.7
	College	805	58.3
	Master’s degree/Doctorate	386	28.0
**Income status**	Income less than expenses	378	27.4
	Income equal to expenses	683	49.5
	Income greater than expenses	320	23.2
**Living arrangement**	Alone	175	12.7
	With family	1189	86.1
	Other (relative/friend)	17	1.2
**Profession**	Physician	322	23.3
	Nurse/midwife	537	38.9
	Other healthcare personnel	522	37.8
**Level of care**	Primary care	255	18.5
	Secondary care	441	31.9
	Tertiary care	685	49.6
**Work schedule**	Day shift	774	56.0
	Shift/on-call	607	44.0
**Chronic disease**	Yes	379	27.4
	No	1002	72.6
**Psychiatric disease**	Yes	126	9.1
	No	1255	89.9

**Figure 1:**
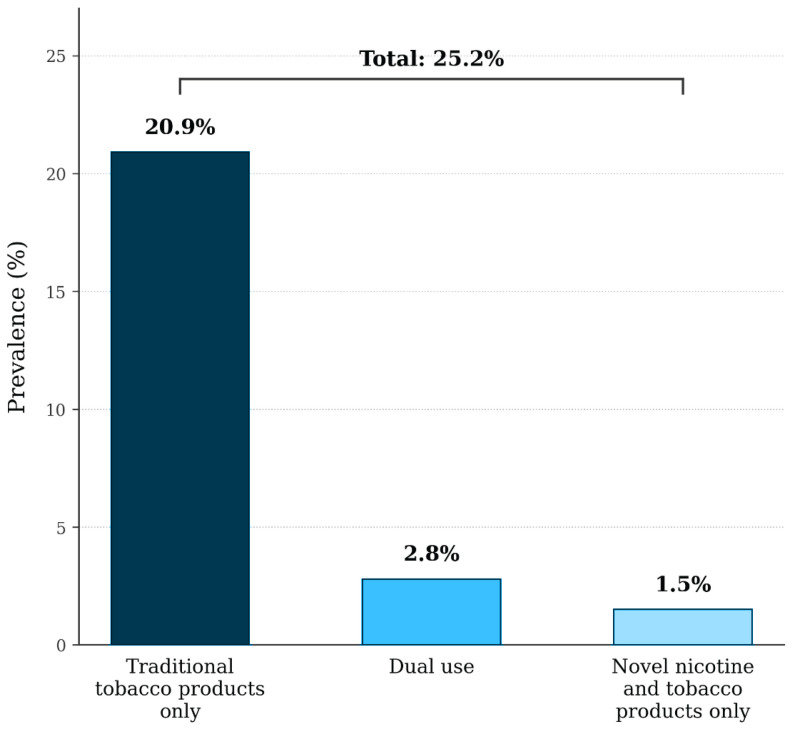
Distribution of current tobacco product use patterns among healthcare workers in a cross-sectional study conducted in public primary, secondary, and tertiary care facilities in Trabzon, Türkiye, July 2025–February 2026 (N=1381)

The prevalence of current tobacco product use was 25.2% (95% CI: 23.0–27.4). Among all participants, 20.9% used traditional tobacco products only, 1.5% used novel nicotine and tobacco products only, and 2.8% reported dual use (Figure 1). The mean age at first tobacco product use among current users was 20.4 ± 5.4 years. In this group, 51.2% reported an intention to quit tobacco products and 46.1% had a previous quit attempt. In addition, 16.5% had received training on tobacco cessation, whereas only 12.0% had received professional cessation support. Regarding nicotine dependence, 50.3% of current tobacco users had low dependence, 37.7% had moderate dependence, and 12.0% had high dependence. The mean FTND total score was 3.4 ± 2.5 (Table 2).

**Table 2:** Prevalence of tobacco use, cessation-related characteristics, and nicotine dependence among healthcare workers in a cross-sectional study conducted in public primary, secondary, and tertiary care facilities in Trabzon, Türkiye, July 2025–February 2026 (N=1381)

*Variables*	*n*	*%*	*95 % CI*
**Tobacco use**			
Current use of any tobacco product	348	25.2	23.0–27.4
**Current type of tobacco used**			
Traditional only	289	20.9	18.9–23.1
Novel nicotine and tobacco product only	20	1.5	0.9–2.0
Dual use	39	2.8	2.0–3.8
**Cessation related characteristics among current tobacco users** (N=348)			
Intention to quit tobacco products (N=344)	176	51.2	45.6–56.1
Previous quit attempt (N=343)	158	46.1	41.1–51.3
Received training on tobacco cessation (N=345)	57	16.5	12.5–20.3
Received professional cessation support (N=343)	41	12.0	8.5–15.7
**Nicotine dependence among current tobacco users** (N=348)			
Low	164	50.3	44.8–55.8
Moderate	123	37.7	32.5–42.9
High	39	12.0	8.6–15.3
**FTND total score**, mean ± SD	3.4 ± 2.5		

FTND: Fagerström test for nicotine dependence.

**Figure 2:**
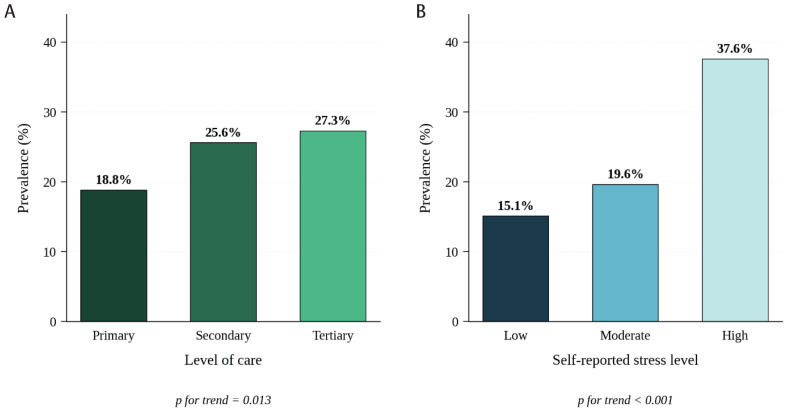
Prevalence of current tobacco product use according to level of care and self-reported stress group among healthcare workers in a cross-sectional study conducted in public primary, secondary, and tertiary care facilities in Trabzon, Türkiye, July 2025–February 2026 (N=1381)

The prevalence of current tobacco product use was 18.8% in primary care, 25.6% in secondary care, and 27.3% in tertiary care institutions (p for trend =0.013). According to the self-reported stress group, the prevalence was 15.1% in the low-stress group, 19.6% in the moderate-stress group, and 37.6% in the high-stress group (p for trend <0.001) (Figure 2).

In the multivariable logistic regression analysis, male sex (adjusted odds ratio, AOR=1.84; 95% CI: 1.33–2.56), older age (AOR=1.03; 95% CI: 1.01–1.05), being single (AOR=1.74; 95% CI: 1.03–2.94), working in secondary care (AOR: 1.69; 95% CI: 1.10–2.60) or tertiary care (AOR=1.58; 95% CI: 1.05–2.38), shift/on-call work (AOR=1.51; 95% CI: 1.12–2.04), having at least one tobacco user in the close environment (AOR=10.40; 95% CI: 5.20–20.81), exposure to pro-tobacco content on social media (AOR=1.73; 95% CI: 1.31–2.27), and higher self-reported stress score (AOR=1.31; 95% CI: 1.22–1.42) were independently associated with current tobacco product use. In contrast, having an income higher than expenses was associated with lower odds of current tobacco product use (AOR=0.54; 95% CI: 0.36–0.82). Profession, education level, have children, living arrangement, and psychiatric illness were not significantly associated with current tobacco product use in the fully adjusted model (p>0.05) (Table 3).

**Table 3:** Multivariable logistic regression analysis of factors associated with current tobacco product use among healthcare workers, by sex, in a cross-sectional study conducted in public primary, secondary, and tertiary care facilities in Trabzon, Türkiye, July 2025–February 2026 (N=1381)

*Variables*	*Total*	*Men*	*Women*
	*AOR (95% CI)*	*AOR (95% CI)*	*AOR (95% CI)*
**Age** (years)	1.03 (1.01–1.05)*	1.02 (0.98–1.05)	1.04 (1.02–1.07)**
**Self-reported stress score**	1.31 (1.22–1.42)***	1.40 (1.22–1.61)***	1.28 (1.17–1.41)***
**Gender**			
Female (ref.)	1		
Male	1.84 (1.33–2.56)***		
**Marital status**			
Married (ref.)	1	1	1
Single	1.74 (1.03–2.94)*	1.19 (0.44–3.25)	2.20 (1.13–4.29)*
**Have children**			
No (ref.)	1	1	1
Yes	1.22 (0.72–2.09)	0.54 (0.20–1.41)	1.80 (0.90–3.59)
**Education level**			
High school/Associate’s degree (ref.)	1	1	1
College	0.81 (0.54–1.20)	0.80 (0.39–1.65)	0.85 (0.52–1.39)
Master’s/Doctorate	0.78 (0.38–1.60)	1.08 (0.24–4.88)	0.77 (0.33–1.78)
**Income status**			
Income lower than expenses (ref.)	1	1	1
Income equal to expenses	0.86 (0.63–1.17)	0.79 (0.43–1.44)	0.92 (0.64–1.32)
Income higher than expenses	0.54 (0.36–0.82)***	0.72 (0.35–1.46)	0.46 (0.27–0.78)**
**Living arrangement**			
Alone (ref.)	1	1	1
With family	0.67 (0.42–1.07)	0.97 (0.41–2.30)	0.55 (0.31–0.97)*
Other (relative/friend)	0.93 (0.29–2.98)	1.90 (0.28–13.00)	0.62 (0.11–3.48)
**Profession**			
Physician (ref.)	1	1	1
Nurse/midwife	0.90 (0.43–1.91)	1.01 (0.18–5.64)	0.65 (0.27–1.58)
Other healthcare personnel	1.29 (0.63–2.64)	2.29 (0.55–9.64)	1.04 (0.43–2.52)
**Level of care**			
Primary (ref.)	1	1	1
Secondary	1.69 (1.10–2.60)*	2.53 (1.18–5.40)*	1.29 (0.76–2.20)
Tertiary	1.58 (1.05–2.38)*	2.33 (1.15–4.73)*	1.23 (0.74–2.05)
**Work schedule**			
Daytime work (ref.)	1	1	1
Shift/on-call work	1.51 (1.12–2.04)***	1.17 (0.69–1.98)	1.82 (1.25–2.65)**
**At least one tobacco user in close environment**			
No (ref.)	1	1	1
Yes	10.40 (5.20–20.81)***	10.77 (3.64–31.85)***	11.54 (4.58–29.04)***
**Psychiatric disease**			
No (ref.)	1	1	1
Yes	1.44 (0.93–2.23)	1.61 (0.63–4.16)	1.31 (0.80–2.17)
**Exposure to pro-tobacco content on social media**			
No (ref.)	1	1	1
Yes	1.73 (1.31-2.27)***	1.73 (1.06–2.83)*	1.67 (1.19–2.33)**

HCWs: healthcare workers. AOR: adjusted odds ratio.

* p<0.05,

** p<0.01,

*** p<0.001.

In sex-stratified analyses, working in secondary care (AOR=2.53; 95% CI: 1.18–5.40), working in tertiary care (AOR=2.33; 95% CI: 1.15–4.73), having at least one tobacco user in the close environment (AOR=10.77; 95% CI: 3.64–31.85), exposure to pro-tobacco content on social media (AOR=1.73; 95% CI: 1.06–2.83), and higher self-reported stress score (AOR= 1.40; 95% CI: 1.22–1.61) were associated with current tobacco product use among men. Among women, older age (AOR=1.04; 95% CI: 1.02–1.07), being single (AOR=2.20; 95% CI: 1.13–4.29), shift/on-call work (AOR=1.82; 95% CI: 1.25–2.65), having at least one tobacco user in the close environment (AOR=11.54; 95% CI: 4.58–29.04), exposure to pro-tobacco content on social media (AOR=1.67; 95% CI: 1.19–2.33), and higher self-reported stress score (AOR=1.28; 95% CI: 1.17–1.41) were associated with current tobacco product use. Having income greater than expenses (AOR=0.46; 95% CI: 0.27–0.78) and living with family (AOR= 0.55; 95% CI: 0.31–0.97) were associated with lower odds among women (Table 3).

**Figure 3:**
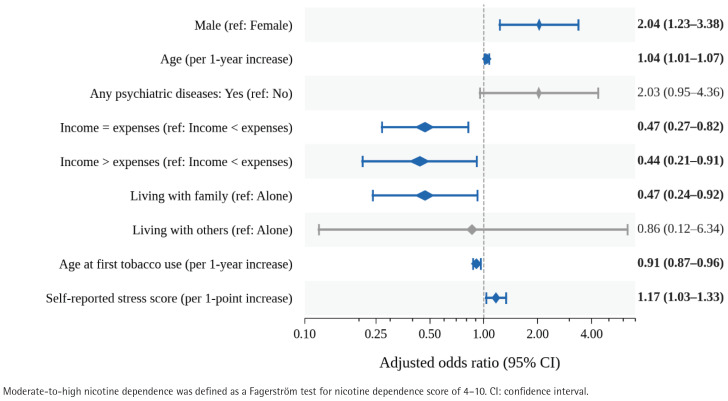
Forest plot of factors associated with moderate-to-high nicotine dependence among current tobacco users among healthcare workers in a cross-sectional study conducted in public primary, secondary, and tertiary care facilities in Trabzon, Türkiye, July 2025–February 2026 (N=348)

Among current tobacco users, male sex compared with female sex (AOR=2.04; 95% CI: 1.23–3.38), older age (AOR=1.04; 95% CI: 1.01–1.07), and higher self-reported stress score (AOR=1.17; 95% CI: 1.03–1.33) were associated with increased odds of moderate-to-high nicotine dependence. In contrast, having income equal to expenses (AOR=0.47; 95% CI: 0.27–0.82) or income greater than expenses (AOR=0.44; 95% CI: 0.21–0.91) compared with income lower than expenses, living with family compared with living alone (AOR=0.47; 95% CI: 0.24–0.92), and older age at first tobacco product use (AOR=0.91; 95% CI: 0.87–0.96) were associated with decreased odds of moderate-to-high nicotine dependence. Psychiatric disease and living with others were not significantly associated with moderate-to-high nicotine dependence (p>0.05) (Figure 3).

## DISCUSSION

In this study, current tobacco product use was observed in one in four healthcare workers, with traditional products predominating, although novel nicotine and tobacco products and dual use were also present. Approximately half of current tobacco users had moderate-to-high nicotine dependence, while the use of professional cessation support was limited. Current tobacco product use was associated with several demographic, occupational, social, and psychosocial factors, including male sex, older age, being single, secondary or tertiary care employment, shift/on-call work, close-environment tobacco use, social media exposure, and higher self-reported stress. These findings provide a framework for interpreting tobacco use among healthcare workers in relation to both individual characteristics and work-related or social contexts.

Regarding prevalence, tobacco use among healthcare workers was 25.2%. Despite this rate being lower than the 28.3% prevalence reported in the general adult population of Türkiye^12^ and the 33.1% prevalence reported in the adult population of Trabzon^22^, it is noteworthy that one in four healthcare workers uses tobacco products. The finding that the prevalence in this study is lower than that of the general population may be attributable to healthcare workers having a higher level of knowledge about the harms of tobacco and adopting more protective health behaviors. However, given that healthcare workers serve as role models for the community and play a key role in smoking cessation counseling, the fact that use persists in this group points to a significant challenge for tobacco control. In the global literature, a systematic review and meta-analysis involving healthcare workers reported a tobacco use prevalence of 21%^4^; in this regard, the rate in this study is slightly above the global average for healthcare workers. Furthermore, the prevalence of novel nicotine and tobacco product use at 4.3% and dual-use prevalence at 2.8% in this study indicates that tobacco use patterns among healthcare workers are not limited to traditional products. The fact that the prevalence of novel nicotine and tobacco product use was reported as 1.2% in the adult population of Trabzon^22^ suggests that these products may represent an emerging concern among healthcare workers.

In the multivariate analysis evaluating the factors influencing tobacco use, the fact that male gender, increasing age, being single, having at least one tobacco user in the immediate environment, and an increase in general stress scores were independently associated with tobacco use indicates that tobacco use among healthcare professionals is shaped not only by individual characteristics but also by social environment and psychosocial factors. Similarly, the strong predictive role of having tobacco users in one’s immediate social circle suggests that tobacco use behavior may be reinforced by social norms and peer influence. However, the confidence intervals for this variable were relatively wide, particularly in the sex-stratified analyses, which may be related to the small number of participants without a tobacco user in their close environment. Therefore, this association should be interpreted with caution, although its direction was consistent across the overall and sex-stratified models. The association between male gender and being unmarried and tobacco use is consistent with a previous study conducted among healthcare professionals^23^. The fact that an increase in stress scores potentially raises the likelihood of use also indicates that tobacco use among healthcare workers cannot be explained solely by a lack of knowledge; coping patterns related to work and living conditions are also significant. The relationship between stress, burnout, workload, and smoking among healthcare workers has been reported previously^24,25^.

Analysis based on gender revealed that factors influencing tobacco use among healthcare workers differed between women and men. In men, working in secondary and tertiary healthcare settings was associated with tobacco use, while this was not significantly the case for women. However, the relatively wide 95% confidence intervals for level of care in the sex-stratified models suggest limited precision of these estimates, possibly due to smaller subgroup sizes. In women, being single, working shifts or on-call duties, and income level were more significantly associated with tobacco use. Furthermore, living with family in women reduced the likelihood of tobacco use, suggesting a protective role for social support^26^. Conversely, the presence of tobacco users in the immediate environment, social media exposure, and high stress levels emerged as common risk factors for both genders. These results indicate that tobacco control approaches for healthcare workers should be planned taking into account both common risks and gender-specific differences.

The identification of shift work and employment at secondary and tertiary care facilities as independent risk factors in this study suggests that institutional and organizational conditions may have a significant impact on tobacco use. The prevalence rates of 18.8% in primary care, 25.6% in secondary care, and 27.3% in tertiary care, along with the continued increase in risk as one moves to secondary and tertiary care, suggest that a heavier patient load, irregular working hours, and a hospital-based work environment may contribute to this situation. The fact that the occupational variable was not found to be significant in the multivariate model suggests that the observed differences may be related not so much to whether individuals are physicians, nurses/midwives, or other healthcare workers, but rather to the work schedule, institutional structure, and operational processes within which these groups operate. Although the literature reports higher smoking rates among certain groups of healthcare workers, these differences appear to diminish when work environment, shift schedules, and organizational stress factors are controlled for^5,27^. Furthermore, the finding that exposure to tobacco-promoting content on social media was found to be a significant independent variable indicates that digital environments have also become an effective exposure arena for healthcare workers. This finding is consistent with a recent study highlighting the relationship between social media exposure and tobacco use^28^.

In this study, the mean Fagerström test score among healthcare workers who use tobacco products was found to be 3.4 ± 2.5. Low-level nicotine dependence was detected in 50.3% of participants, moderate-level in 37.7%, and high-level in 12.0%. This distribution indicates that tobacco use among healthcare workers is not merely an occasional or mild behavior, but that a significant proportion of users exhibit clinically significant nicotine dependence. A recent study conducted among nurses at a tertiary-level university hospital in Türkiye also reported moderate to high levels of nicotine dependence in approximately one-third of smokers^29^. This suggests that the burden of dependence persists across different occupational groups among healthcare workers.

A notable finding was the discrepancy between individuals’ cessation behavior and utilization of professional support. Although approximately half of current tobacco users reported an intention to quit and a substantial proportion had previously attempted to quit, only 12.0% had received professional cessation support. Despite healthcare professionals constituting a demographic generally better positioned than the general population with regard to access to services and information regarding tobacco cessation, the paucity of professional support is noteworthy. The World Health Organization strongly advocates the provision of evidence-based cessation support to all tobacco users. Furthermore, the WHO considers healthcare workers to be both implementers of this process and one of its target groups^30^. The findings of the systematic review demonstrate that cessation interventions targeting healthcare workers can be effective^31^. However, the low rate of professional support observed in this study suggests that the visibility, accessibility, and acceptability of institutional cessation services require improvement.

### Strengths and limitations

A notable strength of this study is its comprehensive sample, which encompasses a diverse range of professional groups engaged in public primary, secondary, and tertiary healthcare institutions within the province of Trabzon. The collaborative evaluation of physicians, nurses/midwives, and other healthcare personnel has facilitated a more comprehensive examination of tobacco product use among healthcare workers. Furthermore, the comprehensive nature of the study’s inquiry, encompassing not only the prevalence of tobacco use but also its intersection with traditional products, novel nicotine and tobacco products, dual use, nicotine dependence, cessation behaviors, social media exposure, and stress, underscores its methodological rigor.

However, the study has some limitations. First, the cross-sectional design prevents any causal inference from the observed associations. Second, the reliance on self-reported questionnaires raises the possibility of recall bias, underreporting, and social desirability bias, especially for sensitive items such as tobacco product use, cessation attempts, and perceived stress. Tobacco use status was not confirmed biochemically, which leaves room for misclassification. Residual confounding remains another concern, since variables we could not measure, like workplace culture, institutional tobacco-control policies, peer norms, and individual coping styles, may have shaped some of the associations we report. The relatively wide confidence intervals seen in certain sex-stratified models also point to limited statistical power within subgroups. Participation was voluntary, and non-participation was not systematically tracked, which may have introduced selection bias. In addition, the sample was drawn from public healthcare institutions in a single province, so the results may not extend to private institutions, other regions, or healthcare workers across Türkiye. Women were also overrepresented in our sample, likely reflecting the higher proportion of female employees in nursing and midwifery; to partially account for this, we ran sex-stratified analyses to see whether the factors linked to tobacco use differed between female and male workers. Multicenter, longitudinal studies covering both public and private institutions will be needed to confirm our findings and to better define the temporal links between occupational, social, and psychosocial factors and tobacco use among healthcare workers.

## CONCLUSIONS

Tobacco use was common among healthcare workers in the study population. Traditional products dominate the pattern of use, but novel nicotine and tobacco products and dual use are also present. Many of the current users showed moderate-to-high nicotine dependence, while only a small proportion had turned to professional support to quit. Tobacco use and nicotine dependence appeared to be shaped by a mix of demographic, occupational, social, and psychosocial factors. Given the cross-sectional design, these relationships cannot be interpreted as causal; rather, they point to associations that may be worth taking into account when planning future research or designing tobacco control and cessation support efforts in this group. Confirming these observations and disentangling their temporal sequence will require multicenter, longitudinal studies.

## Supplementary Material



## Data Availability

The data supporting this research are available from the authors on reasonable request.
