# Supplementary material for: Prevalence of tobacco product use and associated factors among healthcare workers in Trabzon, Türkiye: A cross-sectional study
Source: Tob Induc Dis. 2026 Aug 8;24:10.18332/tid/224356. doi: 10.18332/tid/224356 (PMC13455871; doi:10.18332/tid/224356)
Supplement: Supplementary file 1 [file TID-24-145-s1.pdf]

Tobacco Induced Diseases

Supplementary file

© 2026 Özel M.İ. and Karakullukçu S.

DOI:
10.18332/tid/224356

The content has been provided by the author(s) and has not been reviewed, verified, or endorsed by European Publishing. It may not have undergone peer review. The views, opinions, and recommendations expressed are solely those of the author(s) and do not necessarily reflect the position of European Publishing. European Publishing accepts no responsibility or liability for any consequences arising from the use of, or reliance on, this content.

Supplementary Table S1. Structured questionnaire used in the cross-sectional study conducted among healthcare workers in public primary, secondary, and tertiary care facilities in Trabzon, Türkiye, July 2025–February 2026

The Prevalence of Tobacco Use Among Healthcare Workers in Trabzon Province and Associated Factors – A Cross Sectional Study

1. **What is your age? _______**
2. **What is your gender?** ☐ Female ☐ Male
3. **What is your marital status?** ☐ Single ☐ Married
4. **Your height is _____________** cm
5. **Your weight is ______________** kg
6. **Do you have any children?**

   ☐ No ☐ Yes_______(Please indicate the number.)
7. **What is your level of education? (Please tick the highest qualification you have completed.)**

   ☐ High scholl ☐ Associate degree ☐ Bachelor's degree ☐ Master's degree ☐ PhD/Specialist medical qualification degree
8. **Which of the following best describes your income situation?**

   ☐ My income is less than my expenses

   ☐ My income is equal to my expenses

   ☐ My income is more than my expenses
9. **Who do you live with?**

   ☐ On my own

   ☐ With my family

   ☐ With friends

   ☐ With relatives (excluding spouse, mother, father, and siblings)
10. **Do you have any chronic disease?**

    ☐ Yes ☐ No

11. If your answer is yes, which of the following is your chronic disease? (You may tick more than one option.)

☐ Diabetes ☐ Hypertension

☐ Liver diseases ☐ Lung diseases (such as asthma, bronchitis)

☐ Kidney diseases ☐ Stomach diseases (such as reflux, gastritis)

☐ Musculoskeletal disease ☐ Heart diseases

☐ Thyroid disease

☐ Other: ____________________(Please specify.)

12. Do you have any psychiatric disease?

☐ Yes ☐ No

13. If your answer is yes, which of the following is your psychiatric condition (you may tick more than one option)?

☐ Depression ☐ Bipolar disorder

☐ Panic disorder ☐ Social phobia

☐ Schizophrenia ☐ Generalised anxiety disorder

☐ Attention deficit hyperactivity disorder

☐ Other: ____ (Please specify.)

14. What is your profession?

☐ Specialist Doctor ☐ General Practitioner ☐ Specialist Dentist

☐ General Dentist ☐ Pharmacist ☐ Dietitian

☐ Biologist ☐ Psychologist ☐ Physiotherapist

☐ Nurse ☐ Midwife ☐ Healthcare Technician/Technologist

☐ Medical Secretary

☐ Other: ____ (Please specify.)

15. Place of work:

☐ District Health Directorate (İSM/KETEM/Healthy Living Centre)

☐ Community Health Centre ☐ Family Health Centre

☐ Teaching and Research Hospital ☐ State Hospital

☐ 112 (Ambulance/Emergency Command)

☐ Other: ____ (Please specify.)

16. In which departments do you work? (You may tick more than one option.)

☐ Administrative Unit ☐ Outpatient Department

☐ Outpatient Clinic ☐ Emergency Clinic

☐ Operating Theatre ☐ Intensive Care

☐ Other: ____ (Please specify.)

17. Total length of service in your profession: _______/_________ months/years

18. Total length of service in your current department: _______/_________ months/years

19. Average daily working hours: _______/_________ minutes/hours

20. Which work style suits you best?

☐ Day shift ☐ On-call duty in addition to regular work hours ☐ Shift work

21. Do you use any tobacco or tobacco products?

☐ I have never used any tobacco or tobacco products in my life

☐ I have given up (Please state how many months/years you used the tobacco or tobacco products you have given up.)

☐ Cigarettes: I used them for ……/……… months/years. I used an average of ________ per day.

☐ Roll-up cigarettes: I used them for ……/……… months/years. I used an average of ________ per day

☐ Cigars/Pipes: I used them for ……/……… months/years

☐ Chewing tobacco ……/……… months/years

☐ Hookah ……/……… months/years

☐ Snuff ……/……… months/years

☐ I currently use them

☐ I have been using cigarettes for ……/……… months/years. I used an average of ________ cigarettes per day.

☐ I have been using hand-rolled cigarettes for ……/……… months/years. I use an average of ______ cigarettes per day

☐ I have been using cigars/pipes for ……/……… months/years.

☐ I have been using chewing tobacco for ……/……… months/years.

☐ I have been using Maras herb for ……/……… months/years.

☐ I have been using a hookah for ……/……… months/years.

☐ I have been using snuff for ……/……… months/years.

22. If you use tobacco or tobacco products, what are your reasons for doing so?

☐ To improve my work performance

☐ For enjoyment

☐ Financial difficulties

☐ To fit in with the environment

☐ Pressure from others

☐ Other __________________________(Please specify.)

23. What are your reasons for not using tobacco or tobacco products?

☐ It is harmful to health

☐ Family/cultural background

☐ My religious beliefs

☐ The unpleasant smell

☐ Illness or death in my family or among close relatives linked to tobacco use

☐ It does not interest me

☐ To protect the health of those around me

☐ Because those around me do not smoke

☐ Other __________________________(Please specify.)

24. Do you use any new-generation tobacco or tobacco products?

☐ I have never used any new-generation tobacco or tobacco products in my life.

☐ I have stopped (Please specify how many months/years you used the new-generation tobacco or tobacco products you have stopped using.)

☐ I used an e-cigarette for ……/……… months/years.

☐ I used heated tobacco for ……/……… months/years.

☐ I used a nicotine pouch for ……/……… months/years.

☐ I used Puff for ……/……… months/years.

☐ Other_______________________________________ (Please specify.) ……/……… months/years.

☐ I am currently using

☐ I have been using an e-cigarette for ……/……… months/years.

☐ I have been using heated tobacco for ……/……… months/years.

☐ I have been using nicotine pouches for ……/……… months/years.

☐ I have been using Puff for ……/……… months/years.

☐ Other_______________________________________ (Please specify.) I have been using it for ……/……… months/years.

25. If you use new-generation tobacco or tobacco products, what are your reasons for using them?

☐ To quit traditional tobacco products

☐ Because they are less harmful than traditional tobacco products

☐ Because they look better than traditional tobacco products

☐ To improve my work performance

☐ For enjoyment

☐ Financial difficulties

☐ To fit in with the environment

☐ An offer from others

☐ Other ______________________________________ (Please specify.)

26. What are your reasons for not using new-generation tobacco/tobacco products?

☐ Because they are harmful to health

☐ Family/cultural background

☐ Due to my religious beliefs

☐ Unpleasant smell

☐ Illness or death in my family/close circle linked to tobacco use

☐ It does not interest me

☐ To protect the health of those around me

☐ Because those around me do not use it

☐ Other ____________________________________(Please specify.)

27. At what age did you first use tobacco/tobacco products? _______

28. In which settings do you use tobacco products? (You may tick more than one option.)

☐ At work ☐ At home ☐ In social settings ☐ Other _______________________________(Please specify.)

29. Is there anyone in your immediate circle who uses tobacco or tobacco products? (You may tick more than one option.)

	Yes	No	Left
Husband/Wife/Partner			
Close Friend			
Sibling			
Mother			
Father			
Uncle, Aunt			

30. Are you exposed to offers to use tobacco/tobacco products in your workplace?

☐ Yes, frequently ☐ Yes, occasionally ☐ Yes, rarely ☐ No

31. Please indicate how much time you spend on social media each day.

☐ Less than 2 hours ☐ 2–3 hours ☐ 4–6 hours ☐ More than 6 hours

32. Do you come across content on social media that encourages the use of tobacco or tobacco products (advertisements, influencers, scenes from TV series, etc.)?

☐ Yes ☐ No

33. How often do you come across content on social media that encourages the use of tobacco or tobacco products (advertisements, influencers, TV show scenes, etc.)?

☐ Very often ☐ Occasionally ☐ Rarely

34. How do you find that content relating to tobacco/tobacco product use affects your own tobacco/tobacco product use?

☐ It has no effect ☐ It encourages me to use tobacco/tobacco products

☐ It reduces my desire to quit ☐ It encourages me to use more tobacco/tobacco products

(Please complete this section if you currently use tobacco or tobacco products.)

35. Do you want to give up tobacco products?

☐ Yes ☐ No

36. Have you tried to give up tobacco and tobacco products?

☐ Yes ☐ No

37. Have you received any training on quitting tobacco/tobacco products?

☐ Yes ☐ No

38. Have you sought professional help to quit tobacco and tobacco products?

☐ Yes ☐ No

39. If you have received professional help, through which channels did you receive this help? (You may tick more than one option.)

☐ Alo 171 Smoking Helpline

☐ 115 YEDAM Counselling Helpline

☐ Yeşilay Counselling Centre (YEDAM)

☐ Teaching and Research Hospital

☐ District Health Directorate/Healthy Living Centre

☐ Private healthcare institutions and clinics

☐ University Smoking Cessation Clinic

☐ Other ____ (Please specify.)

40. How soon do you plan to quit tobacco and tobacco products?

☐ I do not plan to quit. ☐ Within 1 month

☐ Within 1–6 months ☐ Within 6–12 months

41. Is there a smoking cessation clinic at your workplace?

☐ Yes ☐ No ☐ I don't know

42. Is there a poster or notice regarding tobacco/tobacco products at your workplace?

☐ Yes ☐ No ☐ I don't know

43. When do you smoke your first cigarette after waking up in the morning?

☐ Within the first 5 minutes of waking up ☐ 6–30 minutes ☐ 31–60 minutes
☐ One or more hours later

44. Do you find it difficult to comply with smoking bans in places such as buses, hospitals or cinemas?

☐ Yes ☐ No

45. At what time of day can you not do without a cigarette?

☐ The first cigarette I smoke in the morning ☐ Any other time

46. How many cigarettes do you smoke during the day?

☐ 10 or fewer ☐ 11–20

☐ 21–30 ☐ 31 or more

47. Do you smoke more frequently in the early hours of the morning compared to other times of the day?

☐ Yes ☐ No

48. Would you smoke even if you were too ill to get out of bed?

☐ Yes ☐ No

49. How would you generally rate your stress levels? (Please rate on a scale of 0 to 10.)

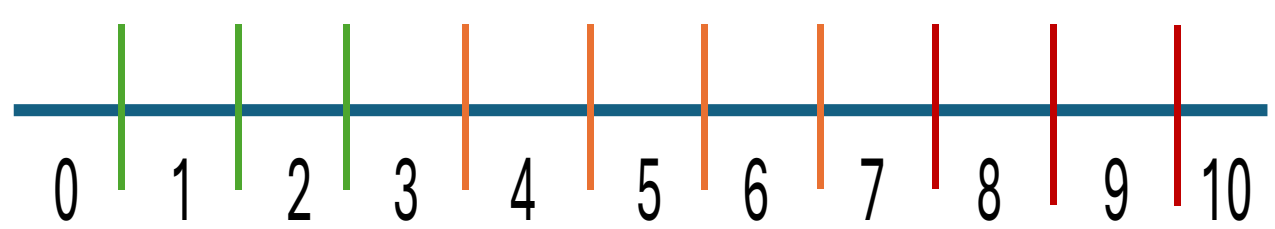

50. Perceived Stress Scale

	Never	Hardly ever	Sometimes	Quite often	Very often
1. Last month, how often did you feel upset because of unexpected events?					
2. Last month, how often did you feel that you couldn't control the important things in your life?					
3. Last month, how often did you feel irritable and stressed?					
4. Last month, how often did you successfully cope with everyday challenges?					
5. Last month, how often did you feel you had effectively dealt with significant changes in your life?					
6. Last month, how often did you feel confident in your ability to handle your personal problems?					
7. Last month, how often did you feel that everything was going well?					
8. Last month, how often did you realise you were unable to cope with the things you needed to do?					

9. Last month, how often were you able to control the difficulties in your life?					
10. Last month, how often did you feel you had managed to cope with everything?					
11. Last month, how often did you get angry because of events that were beyond your control?					
12. Last month, how often did you find yourself thinking about the things you had to achieve?					
13. Last month, how often were you able to control how you spent your time?					
14. Last month, how often did you feel that problems had piled up to the point where you couldn’t cope with them?					

Supplementary Table S2. Univariate logistic regression analysis for factors associated with current tobacco product use among healthcare workers in a cross-sectional study conducted in public primary, secondary, and tertiary care facilities in Trabzon, Türkiye, July 2025–February 2026

**Variables**	**Total HCW**	**Men HCW**	**Women HCW**
	**OR (95% CI)**	**OR (95% CI)**	**OR (95% CI)**
**Age (years)**	**0.996 (0.984-1.009)**	**0.97 (0.95-0.99)****	1.01 (0.99-1.03)
**Self Reported Stress Score**	**1.36 (1.27-1.46)*****	**1.50 (1.32-1.71)*****	**1.31 (1.20-1.43)*****
**Gender**			
Female	Ref		
Male	**1.75 (1.35-2.26)*****		
**Marital Status**			
Married	Ref	Ref	Ref
Single	**1.48 (1.14-1.92)****	**1.78 (1.14-2.77)***	1.31 (0.94-1.82)
**Having Children**			
No	Ref	Ref	Ref
Yes	1.26 (0.98-1.63)	**0.51 (0.33-0.78)****	1.03 (0.75-1.41)
**Education Level**			
High school / Associate's Degree	Ref	Ref	Ref
College	**0.62 (0.44-0.87)****	0.54 (0.29-1.00)	0.68 (0.44-1.03)
Master’s degree / Doctorate	0.73 (0.50-1.07)	0.65 (0.35-1.22)	0.69 (0.42-1.12)
**Income Status**			
Income lower than expenses	Ref	Ref	Ref

Income equal to expenses	0.79 (0.59-1.04)	0.65 (0.39-1.10)	0.81 (0.58-1.13)
Income higher than expenses	**0.55 (0.38-0.78)****	0.59 (0.33-1.05)	**0.42 (0.26-0.67)*****
**Living arrangement**			
Alone	Ref	Ref	Ref
With Family	**0.58 (0.41-0.81)****	**0.55 (0.32-0.96)***	**0.62 (0.40-0.96)***
Other (relative/friend)	1.02 (0.36-2.89)	1.68 (0.35-8.15)	0.59 (0.12-2.92)
**Profession**			
Physician	Ref	Ref	Ref
Nurse/Midwife	0.75 (0.54-1.03)	0.86 (0.35-2.11)	0.95 (0.63-1.44)
Other healthcare personnel	1.08 (0.79-1.47)	1.03 (0.66-1.60)	1.13 (0.72-1.76)
**Level of Care**			
Primary	Ref	Ref	Ref
Secondary	**1.49 (1.02-2.17)***	1.81 (0.95-3.43)	1.39 (0.86-2.22)
Tertiary	**1.62 (1.13-2.31)****	**2.55 (1.41-4.61)****	1.33 (0.85-2.08)
**Work Schedule**			
Daytime work	Ref	Ref	Ref
Shift/on-call work	**1.52 (1.19-1.94)*****	**1.57 (1.03-2.38)***	**1.50 (1.11-2.04)****
**At least one tobacco user in close environment**			
No	Ref	Ref	Ref
Yes	**10.35 (5.25-20.39)*****	**11.58 (4.13-32.49)*****	**10.40 (4.21-25.69)*****
**Psychiatric Disease**			
No	Ref	Ref	Ref
Yes	**1.75 (1.19-2.57)****	2.00 (0.94-4.28)	1.78 (1.13-2.81)

**Exposure to pro-tobacco content on social media**			
No	Ref	Ref	Ref
Yes	**1.89 (1.47-2.43)*** **	**2.31 (1.51-3.53)*** **	**1.78 (1.30-2.43)*** **

HCW: Healthcare Workers, OR: odds ratio, CI: Confidence Interval, *p<0.05, **p<0.01, ***p<0.001

Supplementary Table S3. Univariate logistic regression analysis for factors associated with moderate-to-high nicotine dependence among current tobacco users in a cross-sectional study conducted in public primary, secondary, and tertiary care facilities in Trabzon, Türkiye, July 2025–February 2026

**Variables**	**Total HCW**
	**OR (95% CI)**
**Age (years)**	**1.01 (0.99-1.04)**
**Self Reported Stress Score**	**1.18 (1.05-1.33)** **
**Age at first tobacco use**	**0.92 (0.88-0.96)*** **
**Gender**	
Female	Ref
Male	**2.15 (1.36-3.39)*** **
**Income Status**	
Income lower than expenses	Ref
Income equal to expenses	**0.52 (0.31-0.85)***
Income higher than expenses	0.60 (0.31-1.14)
**Living arrangement**	
Alone	Ref
With Family	0.67 (0.38-1.19)
Other (relative/friend)	1.10 (0.17-7.10)
**Psychiatric Disease**	

No	Ref
Yes	**2.32 (1.15-4.68)***

HCW: Healthcare Workers, OR: odds ratio, CI: Confidence Interval, *p<0.05, **p<0.01, ***p<0.001
